# TPhP exposure disturbs carbohydrate metabolism, lipid metabolism, and the DNA damage repair system in zebrafish liver

**DOI:** 10.1038/srep21827

**Published:** 2016-02-22

**Authors:** Zhongkun Du, Yan Zhang, Guowei Wang, Jianbiao Peng, Zunyao Wang, Shixiang Gao

**Affiliations:** 1 State Key Laboratory of Pollution Control and Resource Reuse, School of the Environment, Nanjing University, Nanjing, 210023 P. R. China

## Abstract

Triphenyl phosphate is a high production volume organophosphate flame retardant that has been detected in multiple environmental media at increasing concentrations. The environmental and health risks of triphenyl phosphate have drawn attention because of the multiplex toxicity of this chemical compound. However, few studies have paid close attention to the impacts of triphenyl phosphate on liver metabolism. We investigated hepatic histopathological, metabolomic and transcriptomic responses of zebrafish after exposure to 0.050 mg/L and 0.300 mg/L triphenyl phosphate for 7 days. Metabolomic analysis revealed significant changes in the contents of glucose, UDP-glucose, lactate, succinate, fumarate, choline, acetylcarnitine, and several fatty acids. Transcriptomic analysis revealed that related pathways, such as the glycosphingolipid biosynthesis, PPAR signaling pathway and fatty acid elongation, were significantly affected. These results suggest that triphenyl phosphate exposure markedly disturbs hepatic carbohydrate and lipid metabolism in zebrafish. Moreover, DNA replication, the cell cycle, and non-homologous end-joining and base excision repair were strongly affected, thus indicating that triphenyl phosphate hinders the DNA damage repair system in zebrafish liver cells. The present study provides a systematic analysis of the triphenyl phosphate-induced toxic effects in zebrafish liver and demonstrates that low concentrations of triphenyl phosphate affect normal metabolism and cell cycle.

Flame retardants are widely used industrial chemicals that are added to many products to prevent them from burning and to reduce the risk of fires. Since 2004, polybrominated diphenyl ethers (PBDEs), such as octaBDE and pentaBDE, which once were used worldwide and which shared a large proportion of the worldwide flame retardant market, have been gradually phased out of the market because of their environmental ubiquity and potential adverse health effects. To satisfy the market demand, production of alternative flame retardants, including organophosphate flame retardants (OPFRs), has been increased^1^. Additionally, OPFRs are physically added but not chemically bonded to materials, which can lead to easy release of such compounds into the environment. Due to their increasing use, OPFRs have been detected in multiple environmental media, including indoor and outdoor air^2^,^3^, surface water^4^, groundwater^5^ and drinking water^6^, and in aquatic biota and human milk^7^. Under this circumstance, the environmental and human health risk assessment of OPFRs is extraordinarily exigent.

Triphenyl phosphate (TPhP, also called TPP) is a high production volume OPFR used in unsaturated polyester resins, in PVC (polyvinyl chloride), and in commercial mixtures, such as FM550 from Chemtura (Firemaster® 550 flame retardant, a Chemtura proprietary mixture of brominated and non-halogen flame retardants) and AC073 from Supresta (commercial flame retardant mixture of proprietary aryl phosphates and triphenyl phosphate)^8^. Due to the increasing use of this compound, TPhP has been the most frequently detected OPFR in both environmental media and biota^9^. According to Zheng *et al.* and Stapleton *et al.*^10^,^11^, TPhP is the main OPFR detected in house dust, with levels of 9810 and 7360 ng/g in China and the USA, respectively. In surface water, the maximum reported level of TPhP was 14000 ng/L in influent samples in Norway in 2007^12^. Although TPhP is easily biodegraded as a non-halogen organophosphate ester and has not been considered to be persistent or bioaccumulative^13^,^14^, the constant and abundant use and release of this compound may also cause sustained exposure to humans and wildlife. Moreover, TPhP has been reported to show multiplex toxicities, including neurotoxicity, developmental toxicity and endocrine disrupting ability, in organisms^15^,^16^,^17^. Various indications have suggested that TPhP poses risks to the environment and to health. However, toxicology data are still insufficient to assess all aspects of the environmental and health risks of TPhP. The liver is a vital organ that plays a major role in metabolism and has numerous functions in vertebrates and is sensitive to xenobiotics. The hepatotoxicity of TPhP remains unknown, although many studies have investigated TPhP metabolism in the liver^18^,^19^,^20^.

Omics, including transcriptomics, proteomics and metabolomics, have been well developed and widely introduced in toxicology studies in the past 10 years^21^. Compared to traditional toxicological endpoints, omics approaches can provide high-throughput data of numerous biomolecules and reflect integrated global responses within an organism^22^. Furthermore, the integration of these omics streams (2 or 3 of them) can provide a deep mechanistic understanding of how compounds perturb normal biological processes and activate defense mechanisms^23^. Deng *et al.* have provided a comprehensive view of the toxic effects of haloacetamides, which are disinfection by-products, by combining metabolomics methods and traditional oxidative stress endpoints^24^. Omics approaches have also been introduced to investigate the toxic effects of OPFRs. Scanlan *et al.*, by using metabolomics and transcriptomics tools, have found that FM550 impairs nutrient utilization or uptake in *Daphnia magna*^25^. Alam *et al.* have investigated the metabolic influences of tributyl phosphate (TBP) and TPhP in a nuclear magnetic resonance (NMR)-based metabolomics study of rat urine and have found that TPhP disturbs cellular energy metabolism and creatine synthesis in rats^26^. These studies have revealed that TPhP or TPhP-containing material impairs nutrient utilization and cellular energy metabolism in different animals at an individual level. Omics is a good methodology to investigate the hepatotoxicity of TPhP.

Zebrafish provide several experimental advantages, such as their low cost, ease of raising large numbers of animals, rapid liver development, and similar molecular and cellular processes to those of humans. Hence, they have been widely employed as a model organism in human disease research and in aquatic ecosystem health studies. The Zebrafish has also been used as a model organism to study drug-induced liver injury^27^. To broaden understanding of the hepatotoxicity of TPhP, we performed transcriptomics and metabolomics analyses of adult zebrafish liver after 7 days of exposure to two different concentrations of TPhP. Histopathological liver changes, blood glucose levels and blood lipid levels were also investigated to provide a systematic understanding of adverse effects induced by TPhP in zebrafish.

## Methods

All experimental protocols were approved by the School of the Environment, Nanjing University, and the methods were carried out in accordance with the approved guidelines.

### Fish Care and Exposure

Five-month-old adult zebrafish (*Danio rerio*, AB strain; sex ratio, 1:1) were purchased from the Institute of Hydrobiology, Chinese Academy of Sciences (Wuhan, China). Before the exposure period, the fish were cultured for more than 1 week in tap water (aerated and basked for two days for dechlorination before use, with oxygen saturation exceeding 80%) and allowed to acclimate to the following conditions^28^: 12h:12h light/dark regime, 26 ± 1 °C, pH 7.4–8.1 and a daily feeding (9:00 am) with dry flake food at a maximum of 2% body weight.

Before exposure experiments were initiated, the 96h-LC_50_ of TPhP to adult zebrafish was determined following the guidance of OECD No. 203^29^ (details are shown in Supplementary information, Section S2). The 96h-LC_50_ of TPhP to adult zebrafish was found to be 1.026 mg/L; thus, the experimental concentrations of TPhP were set at 0.050 and 0.300 mg/L. The concentration 0.050 mg/L was close to the maximum concentration of TPhP that has been reported in surface water. In total, 270 fish were exposed to related concentrations of TPhP (99+%, purchased from Sigma–Aldrich, USA) with a solvent control (0.1% dimethyl sulfoxide [DMSO], chromatographic grade, purchased from Merck, Germany) in 10 L glass tanks. Each tank contained 30 fish (15 males and 15 females), with 3 parallel tanks per treatment group. The exposure experiments were carried out with a semi-static protocol. Half of the solution in each tank was renewed daily. Other conditions were the same as in the above-mentioned acclimation period. The stability of the TPhP concentrations was confirmed using solid phase extraction and high-performance liquid chromatography (details are shown in Supplementary Information, Section S1). The exposure period lasted for 7 days. The fish were fed daily except on the sampling day. When the fish were sampled, they were first anesthetized in ice-cold water.

The exposure experiment was performed twice (540 fish were used), once for metabolomic, transcriptomic and histopathological tests and once for blood and RT-qPCR (reverse transcription-polymerase chain reaction) tests.

### Histopathological Observation

After a one-week exposure period, the livers of zebrafish (10 fish for each concentration group) were dissected and fixed in 10% formalin at 4 °C for 24h. Subsequently, the fixed liver tissues were dehydrated in gradient ethanol, hyalinized in xylene, and embedded in paraffin wax at 56 °C. Then, the paraffin blocks were sectioned at 4-μm thickness. The sections were collected on glass slides and stained with hematoxylin and eosin (H&E) using an H&E Staining Kit (Nanjing Jiancheng Bioengineering Institute, Nanjing, China) following the manufacturer’s recommendations (standard H&E staining protocol). Histologic lesions were observed using an optical microscope equipped with a digital camera.

### Metabolomic Analysis

After a one-week exposure period, the zebrafish were anesthetized on ice, and their livers were dissected and used for extraction immediately. The extraction was carried out using a methanol/chloroform/water system at 4 °C as described by C.Y. Lin *et al.* with minor modifications^30^. In detail, approximately 50 mg frozen liver tissue (dissected from 12 fish) was homogenized using an electric tissue homogenizer in 400 μL methanol (chromatographic grade, Merck, Germany) and 85 μL ultrapure water (UPW) in a glass tube. The homogenizer was washed with 75 μL UPW; this fluid was also collected in the tube. Then, another 200 μL UPW and 400 μL chloroform (chromatographic grade, Merck, Germany) were added after the tube was vortexed for 60 seconds. Next, the tube was centrifuged at 2000 g at 4 °C for 5 min, and 500 μL methanol/water (upper) and 350 μL chloroform (lower) were separately transferred to new glass tubes followed by lyophilization or vacuum drying, respectively. Polar and nonpolar residues were redissolved using 400 μL UPW + 100 μL D_2_O (99.9 atom% D, containing 0.05 wt% TSP, Sigma–Aldrich, USA) or 500 μL chloroform-d (99.8 atom% D, containing 0.03% v/v TMS, Sigma–Aldrich, USA) separately before NMR analysis. Six parallel samples (12 fish were sacrificed for each sample) were prepared for each treatment/control group.

^1^H NMR spectra of all samples were acquired using a Bruker AV600 MHz spectrometer (Bruker Co., Germany) operating at 600.17 MHz and 298.5 K. Spectra of the aqueous samples were acquired using a Carr-Purcell-Meiboom-Gill, 1D acquisition with presaturation (CPMGPR1D) pulse program with 32 scans (FIDs) collected into 65536 data points with Fourier transformation. For the chloroform phase samples, spectra were acquired using a zg pulse program with 16 scans (FIDs) collected into 32768 data points with Fourier transformation.

NMR spectra were processed (phase correction, baseline correction and ppm shift correction using trisilylpropionic acid [TSP] or tetramethylsilane [TMS]) and analyzed (GSD, Global Spectral Deconvolution) using MestReNova v6.1.0–6224 software. Metabolite resonances were identified using both Chenomx NMR Suite 8.02 and published literature^31^,^32^,^33^. NMR spectra and assigned metabolites are presented in Figure S1, Figure S2 and Table S1. Integrations of water resonance (4.70–5.15 ppm in spectra of aqueous samples) and methanol resonance (3.34–3.38 ppm in spectra of aqueous samples and 3.30–3.40 ppm in spectra of chloroform phase samples) were excluded. Following the removal of these regions, the metabolites were then normalized to the total integrated spectral area (0.65–9.37 ppm for aqueous samples and 0.60–7.00 ppm for chloroform phase samples). Then, the data set was log transformed and Pareto scaled (mean-centered and divided by the square root of the standard deviation of each variable) before statistical analysis. Significantly changed metabolites (SCMs) were identified by t-test and partial least squares-discriminant analysis (PLS-DA) using an online tool for metabolomics data analysis, MetaboAnalyst 3.0^34^.

### Transcriptomic Analysis

The livers of 4 zebrafish were dissected and used for total RNA extraction. Total RNA extraction was performed using TRIzol reagent (Life Technologies). Two parallel total RNA samples were prepared for each treatment group. Then, the transcriptomic analysis was conducted by BGI Tech. (Shenzhen, China) using BGI RNA-Seq (Quantification) analysis (http://bgi-international.com/services/genomics/rna-seq-quantification/#tab-id-1). In detail, the total RNA samples were first treated with DNase I to degrade any possible DNA contamination. Then, the mRNA was enriched by using oligo(dT) magnetic beads (for eukaryotes). The mRNA was mixed with the fragmentation buffer and fragmented into short fragments (approximately 200 bp). Then, the first strand of cDNA was synthesized by using random hexamer primers. Buffer, dNTPs, RNase H and DNA polymerase I were added to synthesize the second strand. The double-stranded cDNA was purified with magnetic beads. End repair and 3′-end single nucleotide A (adenine) addition were then performed. Finally, sequencing adaptors were ligated to the fragments. The fragments were enriched by PCR amplification. During the quality control step, an Agilent 2100 Bioanalyzer and ABI StepOnePlus Real-Time PCR System were used to qualify and quantify the sample library. The library products were used for sequencing via an Illumina HiSeq^TM^ 2000 or other sequencer when necessary. Then, the raw data were cleaned by removing low-quality reads as well as reads with adaptor sequences and reads containing unknown bases more than 10%. Clean reads were mapped to reference sequences and/or references. No more than 2 mismatches were allowed in the alignment. Expression levels of the assigned genes were calculated by using the reads per kb per million reads (RPKM) method^35^. Then, differentially expressed genes (DEGs) between TPhP-treated and control groups were screened using the NOIseq method^33^. DEGs were judged by the threshold “false discovery rate (FDR) ≤ 0.001 and the absolute value of log2Ratio ≥1”. Kyoto Encyclopedia of Genes and Genomes (KEGG) pathway enrichment analysis was performed to identify significantly enriched metabolic pathways or signal transduction pathways in DEGs compared with the whole genome background.

### Blood Tests

After the fish were exposed, fish blood samples were collected by tail cutting, as described previously by Chen *et al.*^36^. The tail of each fish was cut off using sterilized spring scissors to expose the caudal vein. Then, blood was collected via pipette (20 μL, Eppendorf, Germany) and transferred into anticoagulant tubes (200 μL, treated with heparin at a final concentration of 20 U/mL blood). Approximately 15 μL blood was collected from each fish; blood from 10 fish (5 males and 5 females) was pooled to provide an adequate plasma volume for subsequent measurements. The collected blood samples were centrifuged at 2000 g at 4 °C for 5 min, and the upper layer (plasma, approximately 50 μL) was collected and stored at −20 °C until use. Three parallel samples were prepared for each treatment group. The glucose, pyruvate, triglyceride (TG), total cholesterol (TC), high-density lipoprotein cholesterol (HDL-C) and low-density lipoprotein cholesterol (LDL-C) contents in the plasma were measured using a glucose assay kit, pyruvate assay kit, TG assay kit, TC assay kit, HDL-C assay kit, and LDL-C assay kit, respectively. All these assay kits were purchased from Nanjing Jiancheng Bioengineering Institute (Nanjing, China).

### RT-qPCR

The expression levels of 6 DEGs that were identified in the transcriptomic analysis were measured by RT-qPCR to verify the reliability of the transcriptomic analysis. Three parallel total RNA samples (4 fish each sample) were prepared for each group with the above-described method. The primer sequences are shown in Table S4. RT-qPCR experiments were performed using a RevertAid First Strand cDNA Synthesis Kit and Maxima SYBR Green/ROX qPCR Master Mix (Thermo Scientific, USA) on a Bio-Rad CFX Connect Real-Time PCR Detection System.

### Statistical Analysis

In the metabolomic analysis, statistical analysis was performed using an online tool, MetaboAnalyst 3.0. SCMs were identified by *p* < 0.05 in t-tests and by a variable influence on projection (VIP) score of >1 in the PLS-DA analysis by comparing the metabolomic profiles between control and TPhP-treated groups. In the transcriptomic analysis, statistical analysis was also conducted by BGI Tech following their standard method of RNA-Seq analysis. For blood tests, the data are expressed as the means ± standard deviation (SD); the blood test data were analyzed with SPSS 16.0 software (SPSS Inc., Chicago, IL, USA). Significant differences between different treatment groups were identified on the basis of *p* < 0.05 using one-way ANOVA followed by post hoc test based on least significant difference (LSD).

## Results

### Metabolomic Alterations

**Figure 1:**
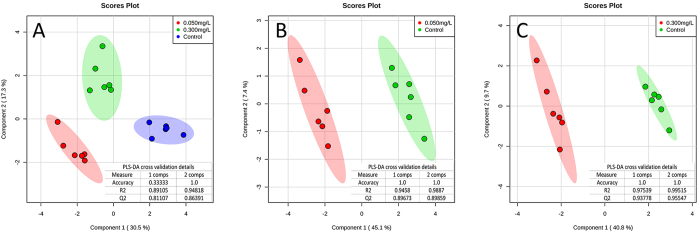
Score plots of PLS-DA models. PLS-DA analyses were performed using MetaboAnalyst 3.0. PLS-DA model among control and 0.050 and 0.300 mg/L TPhP-treated groups (**A**); PLS-DA model between control and 0.050 mg/L TPhP-treated groups (**B**); PLS-DA model between control and 0.300 mg/L TPhP-treated groups (**C**). The shaded areas are the 95% confidence regions of each treatment. Six parallel metabolomics samples were prepared in each treatment group.

The metabolomic analysis of the toxic effects of TPhP in zebrafish liver was performed based on ^1^H NMR. After we obtained the metabolomic profiles of different samples in the control and TPhP-treated (0.050 mg/L, 0.300 mg/L) groups, PLS-DA analysis was performed for pattern recognition (Fig. 1,A). The clear separation of samples from different treatment groups in the score plot of the PLS-DA model indicated that TPhP induced significant changes in zebrafish liver metabolomics. Moreover, samples in 0.050 mg/L and 0.30 mg/L TPhP-treated groups were located at approximately the same area on component 1, indicating that metabolomic changes were similar in these two groups and further confirming that TPhP exposure caused specific metabolomic changes in zebrafish liver. Profiles of samples from 0.050 mg/L and 0.300 mg/L TPhP-treated groups were separately compared with that of the control, and SCMs were identified based on the following criteria: VIP score >1 in the PLS-DA analysis (Fig. 1B,C) and *p* < 0.05 in t-tests, as listed in Table 1.

As shown in Table 1, 19 SCMs were significantly changed due to TPhP exposure; these metabolites were involved in carbohydrate metabolism (glucose, UDP-glucose, glycolate, fumarate, succinate, and lactate), lipid and fatty acid metabolism (choline, acetylcarnitine, esterified cholesterol, arachidonic acid [ARA], timnodonic acid [EPA], linoleic acid and fatty acids identified by αH_2_), amino acid metabolism (glutamate, glutamine and leucine), and osmolyte metabolism (TMAO, dimethylamine [DMA]). These altered metabolites indicated that TPhP induced metabolic disruptions in zebrafish liver.

### Transcriptomic Alterations

**Figure 2:**
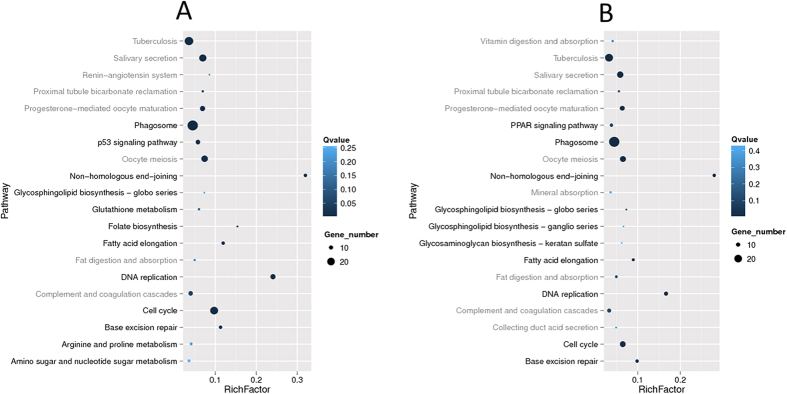
Top 20 influenced pathways of each TPhP-treated group in the transcriptomic analysis. (**A**) Top 20 statistics of KEGG pathway enrichment for DEGs observed in the 0.050 mg/L TPhP-treated group; (**B**) Top 20 statistics of KEGG pathway enrichment for DEGs observed in the 0.300 mg/L TPhP-treated group.

Transcriptional analysis identified 471 and 364 DEGs in 0.050 mg/L and 0.300 mg/L TPhP-treated groups, respectively. The expression levels of 6 DEGs were measured via RT-qPCR to verify the reliability of the transcriptomic analysis. The RT-qPCR results were consistent with those of the transcriptomic analysis (Figure S5), thus validating the transcriptomic analysis. KEGG pathway analysis based on the DEGs was also conducted; the results are listed in Fig. 2 (statistical details are shown in Table S2 and Table S3).

As shown in Fig. 2, most affected pathways were similar for the 0.050 and 0.300 mg/L TPhP-treated groups. TPhP exposure primarily affected the expression of genes related to carbohydrate and lipid metabolism and to the DNA damage repair system in zebrafish. For example, the altered folate biosynthesis pathway (0.05 mg/L group only), amino sugar and nucleotide sugar metabolism pathway (0.05 mg/L TPhP-treated group only), and glycosphingolipid biosynthesis pathway are related to carbohydrate metabolism. The altered fatty acid elongation pathway and PPAR signaling pathway (0.300 mg/L TPhP-treated group only) are related to lipid metabolism. DNA damage repair and cell cycle pathways, including DNA replication, cell cycle, non-homologous end-joining (NHEJ) and base excision repair (BER) pathways, were strongly affected (with large gene numbers and rich factors in Fig. 2; detailed data are shown in Table S2 and S3).

### Blood Tests

**Figure 3:**
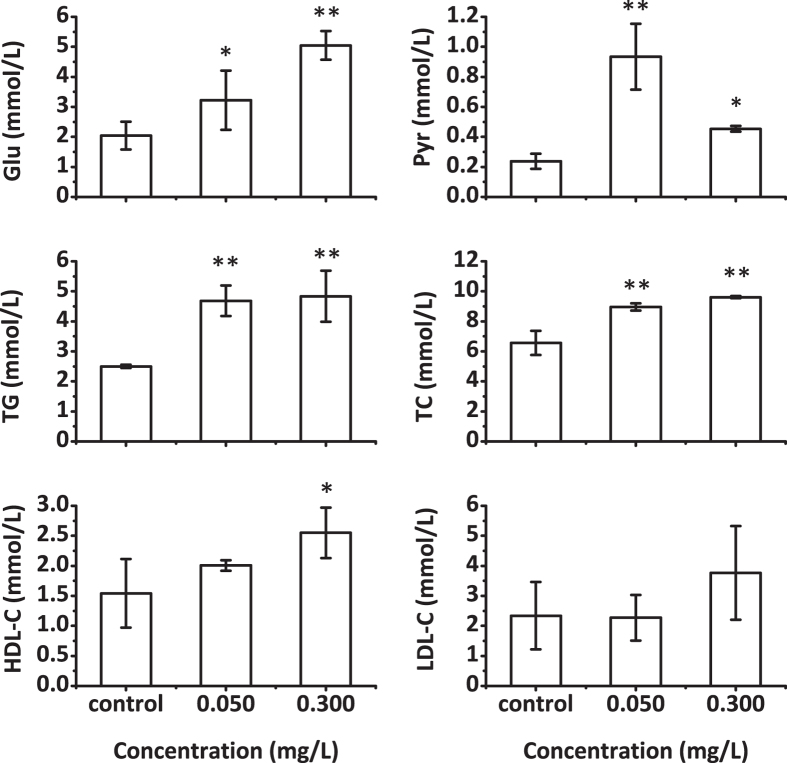
Effects of TPhP exposure on zebrafish blood glucose and lipid levels. The data are presented as the mean ± SD (n = 3). Asterisk (*) and double asterisks (**) denote significant changes identified by one-way ANOVA and post hoc test based on LSD (*p* < 0.05 and *p* < 0.01, respectively) relative to controls.

The blood test results are shown in Fig. 3. After the fish were exposed, blood glucose, pyruvate, triglyceride and total cholesterol levels were significantly elevated in both the 0.050 and 0.300 mg/L TPhP-treated groups. HDL-C levels increased in the 0.300 mg/L TPhP-treated group but showed no statistical change in the 0.050 mg/L TPhP-treated group. LDL-C levels did not significantly change in both the 0.050 and 0.300 mg/L TPhP-treated groups. Blood glucose levels showed a greater increase in the 0.300 mg/L TPhP-treated group than in the 0.050 mg/L TPhP-treated group. In contrast, blood pyruvate levels in 0.050 mg/L TPhP-treated group were higher than were those of the 0.300 mg/L TPhP-treated group.

### Histopathological Liver Changes

**Figure 4:**
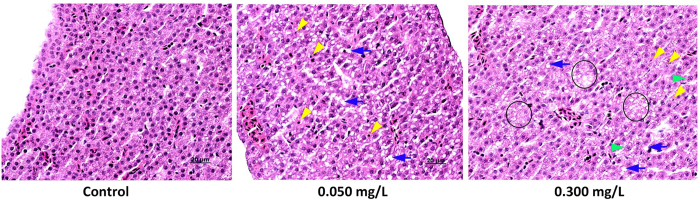
Representative micrographs of liver sections from zebrafish (n = 10) exposed to the indicated experimental concentrations of TPhP for 7 days. Blue arrows indicate vacuolization, yellow arrowheads indicate pyknotic nucleus, green arrowheads indicate karyorrhexis, and areas in black ellipses indicate cells losing nuclei.

Representative histological sections from zebrafish exposed to various concentrations of TPhP are shown in Fig. 4. Compared to the control group, zebrafish liver sections from both the 0.050 and 0.300 mg/L TPhP-treated groups showed obvious vacuolization (blue arrows) and enlarged sinusoidal vessels, indicating lipid accumulation. Pyknotic nuclei (yellow arrowheads) were also found in the TPhP-treated groups, indicating that TPhP induced apoptosis in zebrafish liver. Zebrafish livers in the 0.300 mg/L TPhP-treated group exhibited more serious apoptosis than did those in the 0.050 mg/L TPhP-treated group as indicated by the appearance of karyorrhexis and cells losing nuclei.

## Discussion

The environmental occurrence and concentration of TPhP has increased along with the increasing utilization of TPhP an alternative to PBDEs, as a plasticizer and flame retardant. Environmental and health risk assessments of TPhP are exigent. Existing data have indicated that TPhP causes neurotoxicity, developmental toxicity, and endocrine and metabolic disruption in organisms. Data related to the liver toxicity of this compound are lacking. In the present study, we performed an integrated investigation of the toxic effects induced by TPhP in zebrafish liver by combining metabolomics, transcriptomics, blood tests and histopathological approaches. Combining metabolomics and transcriptomics approaches helped us to confirm our findings by mutual corroboration and to increase our understanding of the mechanism of TPhP-induced hepatotoxicity by connecting bioresponses at different levels.

Based on metabolomic analysis, 15 and 16 SCMs were identified in the 0.050 mg/L and 0.300 mg/L TPhP-treated groups, respectively. Twelve identified SCMs showed the same trend in both treatment groups. Based on the transcriptomic analysis, 301 identical DEGs were identified, and all showed the same trend in both the 0.050 mg/L (number of DEGs: 471) and 0.300 mg/L (number of DEGs: 364) TPhP-treated groups. The results showed high consistency between liver responses under 0.050 mg/L and 0.300 mg/L TPhP exposure, indicating that TPhP exposure had specific toxic effects in zebrafish liver and that these effects were induced at a low TPhP concentration. Based on the multiple tests and analyses, TPhP showed pronounced effects on liver carbohydrate metabolism and lipid and fatty acid metabolism and hampered the DNA repair system in liver cells of zebrafish.

For carbohydrate metabolism, glucose accumulated in the liver and blood of both the 0.050 mg/L and 0.300 mg/L TPhP-treated groups, indicating that glucose utilization might have been hampered. Hampered glucose utilization might be attributed to abnormal insulin secretion and insulin resistance because the expression levels of genes involved in insulin secretion or insulin resistance, such as CFTR8, ins, and cyp17a1 (Table S6, details of the particular genes mentioned below are found in Table S6), were found to be disturbed in the TPhP-treated groups^37^,^38^. The decreased fumarate, lactate and succinate contents (although changes in lactate and succinate were not statistically significant in the 0.300 mg/L TPhP-treated group) suggested that the progression of the TCA cycle and glycolysis was attenuated. Although pyruvate was not identified in the liver metabolomic profiles, the accumulation of pyruvate in the blood might be ascribed to the weaken progression of the TCA cycle and glycolysis. TPhP-induced TCA cycle disturbances in rat and *Daphnia magna* have also been reported in previous studies based on urinary or whole-body metabolomics studies^25^,^26^. Considering these reports, the results of the present study demonstrate that TCA cycle disturbance may be a general TPhP-induced effect on cell metabolism in various organisms. Abnormal UDP-glucose and glycolate contents in TPhP-treated groups (except glycolate in the 0.050 mg/L TPhP-treated group) reflected a disorder of oligosaccharide metabolism. In addition, related genes (Figure S6) and pathways involved in carbohydrate metabolism, such as glycosphingolipid biosynthesis, folate biosynthesis, amino sugar and nucleotide sugar metabolism pathways, were affected by TPhP exposure, as revealed by transcriptomic analysis.

For lipid and fatty acid metabolism, the elevated plasma TG and TC levels and the emergence of large lipid droplets in the livers of TPhP-treated groups suggested that TPhP induced lipid metabolism disorders. In the liver metabolomic profiles of TPhP-treated groups, the levels of metabolites such as choline, acetylcarnitine, esterified cholesterol, fatty acid (linoleic acid, αH2), and phosphatidylserine were significantly decreased, whereas the levels of ARA and EPA were increased. Choline and acetylcarnitine play important roles in promoting liver lipid transfer and utilization and are always used as biomarkers in lipid metabolism studies^39^,^40^. Thus, the reduction of choline and acetylcarnitine might be associated with the emergence of lipid droplets in liver and with elevated plasma TG and TC levels. Decreased choline has also been found in *Daphnia magna* exposed to FM550^25^. The liver is an essential organ in cholesterol synthesis and metabolism. Plasma cholesterol markedly increased after TPhP exposure, whereas no obvious changes in liver cholesterol were observed. This discrepancy might be attributed to inhibition of cholesterol utilization in other organs or might be associated with the reported TPhP-induced disruption of endocrine signaling, as reported previously^41^,^42^. The increase in ARA + EPA and the affected phagosome pathway might indicate that an inflammatory response exists in the TPhP-treated zebrafish because ARA and EPA are inflammatory biomarkers^43^,^44^. However, no obvious inflammatory response was found upon histopathology observation. Further studies are required to determine the reason for this discrepancy. The decrease in linoleic acid, the precursor of ARA, might be attributed to the elevated ARA content^45^. Based on transcriptomic and KEGG pathway analyses, lipid metabolism pathways, such as the fatty acid elongation pathway, were significantly affected by TPhP exposure. Moreover, the PPAR signaling pathway was significantly affected in the 0.300 mg/L and 0.050 mg/L TPhP-treated groups; genes involved in the PPAR signaling pathway also presented abnormal expression profiles. Because the PPAR signaling pathway plays important roles in lipid metabolism and adipocyte differentiation the abnormal PPAR signaling induced by TPhP clearly indicates disturbed lipid metabolism in zebrafish liver. Details of the pathways and DEGs involved in lipid metabolism are found in Figure S6.

In addition to metabolism disturbance, transcriptomic analysis suggested that TPhP exposure hampered the DNA repair system in zebrafish liver cells because pathways such as the NHEJ, BER, DNA replication and cell cycle pathways were strongly affected in both TPhP-treated groups. Because NHEJ eliminates DNA double-strand breaks and BER repairs small base lesions derived from oxidation and alkylation damages, these pathways are key to DNA damage repair, thereby facilitating the maintenance of genome integrity. In TPhP-treated groups, the decreased expression of some genes in these two pathways might reflect a decrease in the DNA repair capacity in zebrafish liver cells, which would induce DNA damage and even apoptosis. In addition, the p53 signaling pathway was affected in the 0.050 mg/L TPhP-treated group. The p53 signaling pathway is a stress response pathway that can be induced by a number of stress signals, including DNA damage, oxidative stress and activated oncogenes. Although the p53 signaling pathway was not listed in the top 20 affected KEGG pathways in the 0.300 mg/L TPhP-treated group (Fig. 2B), key genes involved in this pathway, including p53R2 and Gadd45, were significantly down-regulated. The disturbed p53 pathway was also evidence for TPhP-induced DNA damage in zebrafish liver cells, which was consistent with the apoptosis observed in the histopathological analysis.

In addition to the strong influence of TPhP on carbohydrate metabolism, lipid and fatty acid metabolism and the DNA repair system, changes in glutamate, glutamine, alanine, leucine, TMAO and DMA contents in the metabolomic profile indicated that TPhP also affected amino acid metabolism and osmolyte metabolism in zebrafish liver cells. Amino acid metabolism disturbance induced by the TPhP containing mixture FM550 has also been found in *Daphnia magna*, including changes in amino acids, including glutamine and leucine^25^. The different changes in amino acids between these two studies might be attributed to the different animals and toxicants used. The decreased TMAO and increased DMA levels observed in TPhP-treated groups reveal promotion of TMAO degradation. The decreased choline and fmo5 expression might also reduce TMAO synthesis in liver^46^. As an important osmolyte in fish^47^, the decreased TMAO level suggested that TPhP might also affect the osmotic equilibrium in zebrafish liver.

**Figure 5:**
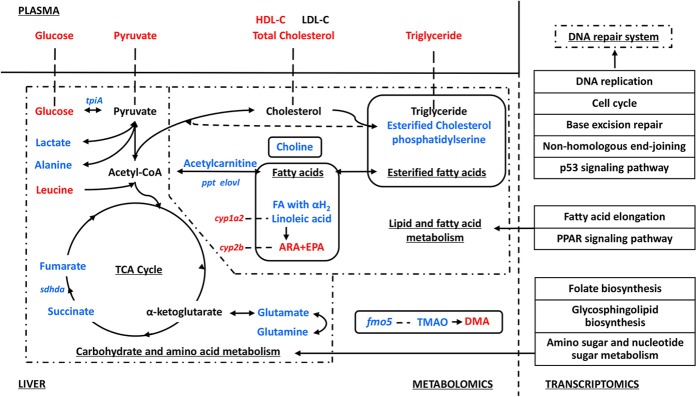
Systematic view of TPhP-induced hepatotoxicity in zebrafish. Red and blue denote up-regulated and down-regulated, respectively.

A systematic view of TPhP-induced effects in zebrafish liver is shown in Fig. 5.

In summary, the present study provides a systematic view of the toxic effects of TPhP in zebrafish liver. These results suggested that TPhP had comprehensive toxic effects in zebrafish liver after a one-week exposure period, even at a low dose (1/20 LC50). Disturbed carbohydrate, lipid, and amino acid metabolism pathways in zebrafish liver indicated that TPhP induced comprehensive metabolic disorders in zebrafish liver and even the whole body. By combining with the previous studies, elevated glucose and decreased choline can be used as potential biomarkers of TPhP exposure. DNA repair systems and the osmotic equilibrium in zebrafish cells were also hampered by TPhP exposure. Although no visible effects on the fish, such as their feeding and activity, were observed, potential consequences of the internal effects induced by chronic exposure to TPhP should be further investigated. Studies also need to be performed to understand the effects of TPhP on DNA damage, osmotic equilibrium and related mechanisms. The hepatotoxicity of other increasingly used OPFRs should also be determined.

## Additional Information

**How to cite this article**: Du, Z. *et al.* TPhP exposure disturbs carbohydrate metabolism, lipid metabolism, and the DNA damage repair system in zebrafish liver. *Sci. Rep.* **6**, 21827; doi: 10.1038/srep21827 (2016).

## Supplementary Material

Supplementary Information

## Figures and Tables

**Table 1:** SCMs in liver induced by TPhP.

	SCMs	0.050 mg/L		0.300 mg/L	
		Fold Change	VIP score	Fold Change	VIP score
carbohydrate metabolism	Fumarate	0.73*	1.07	0.69*	1.23
	Glucose	1.41**	1.23	1.39*	1.26
	Glycolate	0.88	0.58	0.57**	1.63
	Lactate	0.68**	1.32	0.86	0.66
	Succinate	0.68*	1.15	0.75	0.90
	UDP-glucose	0.57**	1.66	1.21*	0.84
lipid and fatty acid metabolism	Choline	0.55**	1.77	0.80**	1.04
	Acetylcarnitine	0.68**	1.32	0.73**	1.22
	Esterified Cholesterol	0.64**	1.43	0.55**	1.76
	FA-ARA+EPA	1.44**	1.31	1.21*	0.83
	FA-LINOLEIC	0.81*	0.89	0.76**	1.11
	FA-αH_2_	0.72**	1.24	0.75*	1.09
	Phosphatidylserine	0.52*	1.64	0.31**	2.48
amino acid metabolism	Alanine	0.43**	2.06	0.60**	1.54
	Glutamate	0.66**	1.41	0.67**	1.43
	Glutamine	0.64**	1.41	0.73*	1.04
	Leucine	1.77**	1.62	1.99**	1.85
TMAO metabolism	Dimethylamine	1.88**	1.72	2.89**	2.46
	Trimethylamine-N-oxide (TMAO)	0.90	0.49	0.56**	1.65

SCMs were identified by performing PLS-DA analysis and t-tests between TPhP-treated groups and the control group (n = 6) using MetaboAnalyst 3.0. VIP scores were obtained from PLS-DA models with a threshold of 1.0. Asterisk (*) and double asterisks (**) denote significant treatment effects identified by Student’s t-test (*p* < 0.05 and *p* < 0.01, respectively) relative to controls. The fold change was obtained by comparing relevant metabolites in the TPhP groups with the control group. SCMs in liver induced by TPhP.
