# Supplementary material for: TPhP exposure disturbs carbohydrate metabolism, lipid metabolism, and the DNA damage repair system in zebrafish liver
Source: Sci Rep. 2016 Feb 22;6:21827. doi: 10.1038/srep21827 (PMC4761896; doi:10.1038/srep21827)
Supplement: Supplementary Information [file srep21827-s1.pdf]

SUPPLEMENTARY INFORMATION

TPhP disturbed carbohydrate metabolism, lipid metabolism, and DNA damage repair system in zebrafish liver.

Zhongkun Du, Yan Zhang, Guowei Wang, Jianbiao Peng, Zunyao Wang, Shixiang Gao*
State Key Laboratory of Pollution Control and Resource Reuse, School of the Environment,
Nanjing University, Nanjing, Jiangsu 210023, China

This Supplementary Information contains:

Section S1. TPhP concentration measurement during the exposure period.
Section S2. Acute toxicity test.
Figure S1. ^{1}H NMR spectra of liver aqueous extracts.
Figure S2. ^{1}H NMR spectra of liver chloroform extracts.
Table S1. List of metabolites assigned from NMR spectra.
Table S2. List of TOP 20 altered KEGG pathways by 0.050 mg/L TPhP exposure identified from transcriptomics analysis.
Table S3. List of TOP 20 altered KEGG pathways by 0.300 mg/L TPhP exposure identified from transcriptomics analysis.
Table S4. Gene-specific primer sequences used for RT-qPCR.
Table S5. RT-qPCR validation of changes in selected genes identified by RNA-Seq.

Section S1. TPhP concentration measurement during the exposure period.

Stability of TPhP concentrations during the semi-static exposure period was assessed. TPhP solutions were sampled (100 mL) at 0 h (the beginning of the exposure period), 24 h (before solution renewal and immediately after solution renewal). Three parallel samples were prepared in each treatment group. TPhP extraction was performed using the solid phase extraction (SPE) method. First, a 100 mL water sample was passed through a Poly-Sery PSD cartridge (250 mg, 6 mL, ANPEL Scientific Instrument, Shanghai). The cartridges concentrated with TPhP were then connected to the drying tube and eluted with 3 mL ethyl acetate. The extracts were concentrated with a nitrogen evaporation apparatus and redissolved using 1 mL (0.050 mg/L group) or 3 mL (0.300 mg/L group) methanol. Then, TPhP concentrations were measured with a HPLC apparatus equipped with an Agilent Eclipse XDB-C18 column (250 × 4.6 mm, 5-µm particle size) and a UV detector (Agilent 1200). The HPLC analysis method: the mobile phase consisted of acetonitrile–water (85:15), the flow rate was 800 µL/min, the injection volume was 20 µL, the column temperature was 30 °C, and the detective wavelength was 210 nm. The retention time of TPhP was 6.82 min, and the recoveries of 0.050 mg/L and 0.300 mg/L samples were 96.1 ± 4.3% and 93.6 ± 5.3%, respectively. The results are shown in the table below. The results suggested that the TPhP concentration did not decrease considerably after 24 h under the experimental conditions and that the semi-static exposure protocol maintained a relatively stable TPhP concentration during the 7-day exposure period.

Group	0h	24h (before solution renewal)	24h (After solution renewal)
0.050 mg/L	0.048 ± 0.002 mg/L	0.042 ± 0.006 mg/L	0.045 ± 0.005 mg/L
0.300 mg/L	0.281 ± 0.016 mg/L	0.248 ± 0.012 mg/L	0.262 ± 0.012 mg/L

Section S2. Acute toxicity test.

An acute toxicity test was performed following the guidance of OECD NO. 203. After the acclimation period, vigorous and healthy adult zebrafish were selected and were randomly divided into the exposed groups and control group. Based on the preliminary experiment, TPhP was diluted into the following concentrations: 0.40, 0.50, 0.80, 1.00, 1.20, 1.50, 1.80, and 2.00 mg/L. Two liter beakers were used as the exposure containers, and each beaker contained ten adult zebrafish. Each test concentration was replicated three times. To keep exposure concentrations and water quality consistent, the exposure solutions were renewed every 24 h. External conditions during the exposure period were identical to the conditions during culture as previously mentioned. The number of dead zebrafish was calculated at 96 h. The standard of determining death was no visible breathing or no movement when the tail was touched. During the acute toxicity experiment, neither food nor aeration was provided, and dead individuals were removed immediately from the exposure solutions. The 96 h-LC_{50} values were calculated using probit regression with $p < 0.05$. The 96 h-LC_{50} value was 1.026 mg/L, with a 95% confidence limit of 0.921 – 1.155 mg/L. The raw data are shown below.

TPhP concentration (mg/L)	Total Fish			Death			Mortality (%)
	1	*2*	*3*	*1*	*2*	*3*	
0.400	10	10	10	0	1	1	6.7 ± 5.8
0.523	10	10	10	1	1	2	13.3 ± 5.8
0.683	10	10	10	2	3	2	23.3 ± 5.8
0.894	10	10	10	3	2	4	30.0 ± 10.0
1.170	10	10	10	6	7	4	56.7 ± 15.3
1.529	10	10	10	6	7	8	73.3 ± 10.0
2.000	10	10	10	9	10	10	96.7 ± 5.8

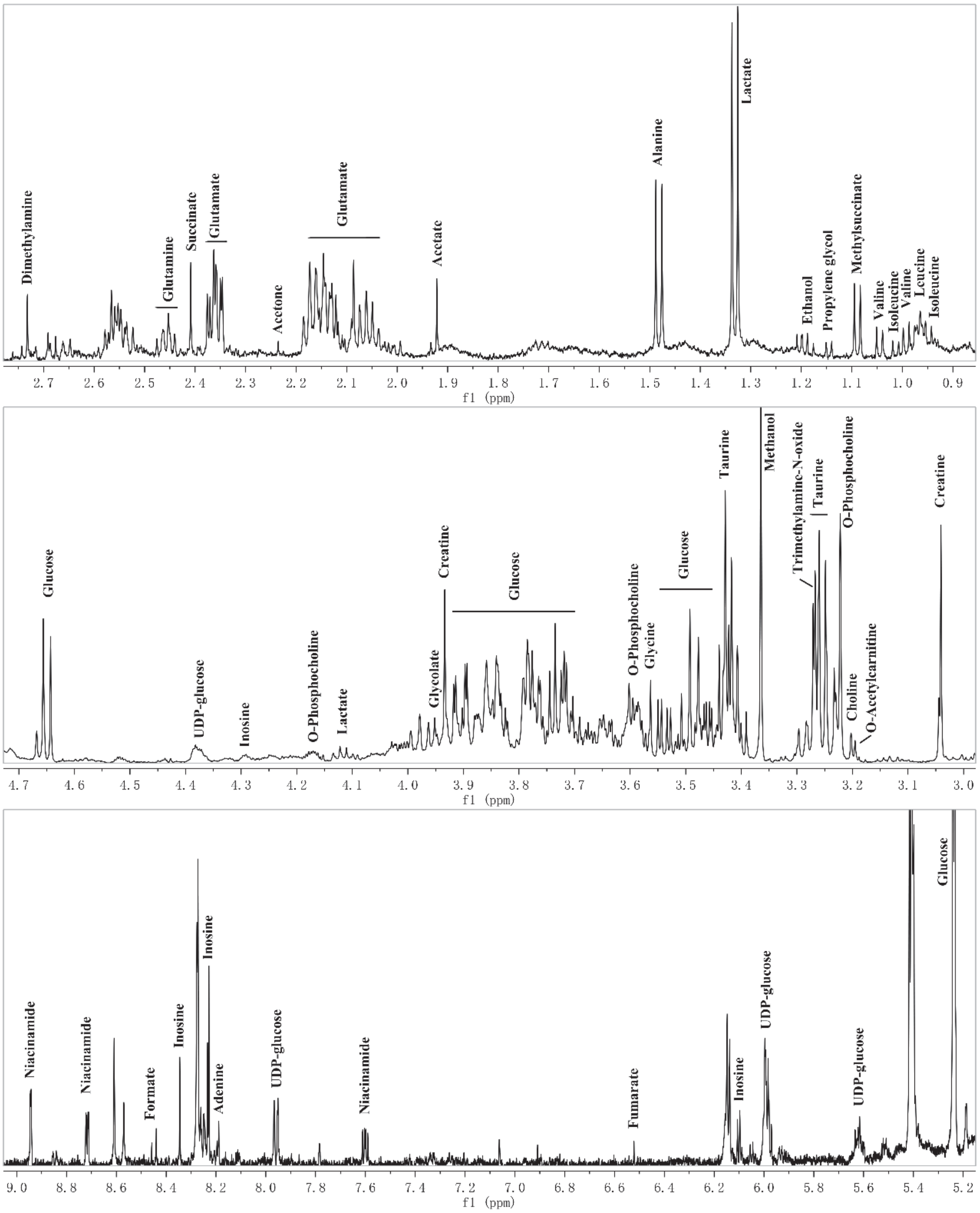

Figure S1. ^{1}H NMR spectra of liver aqueous extracts.

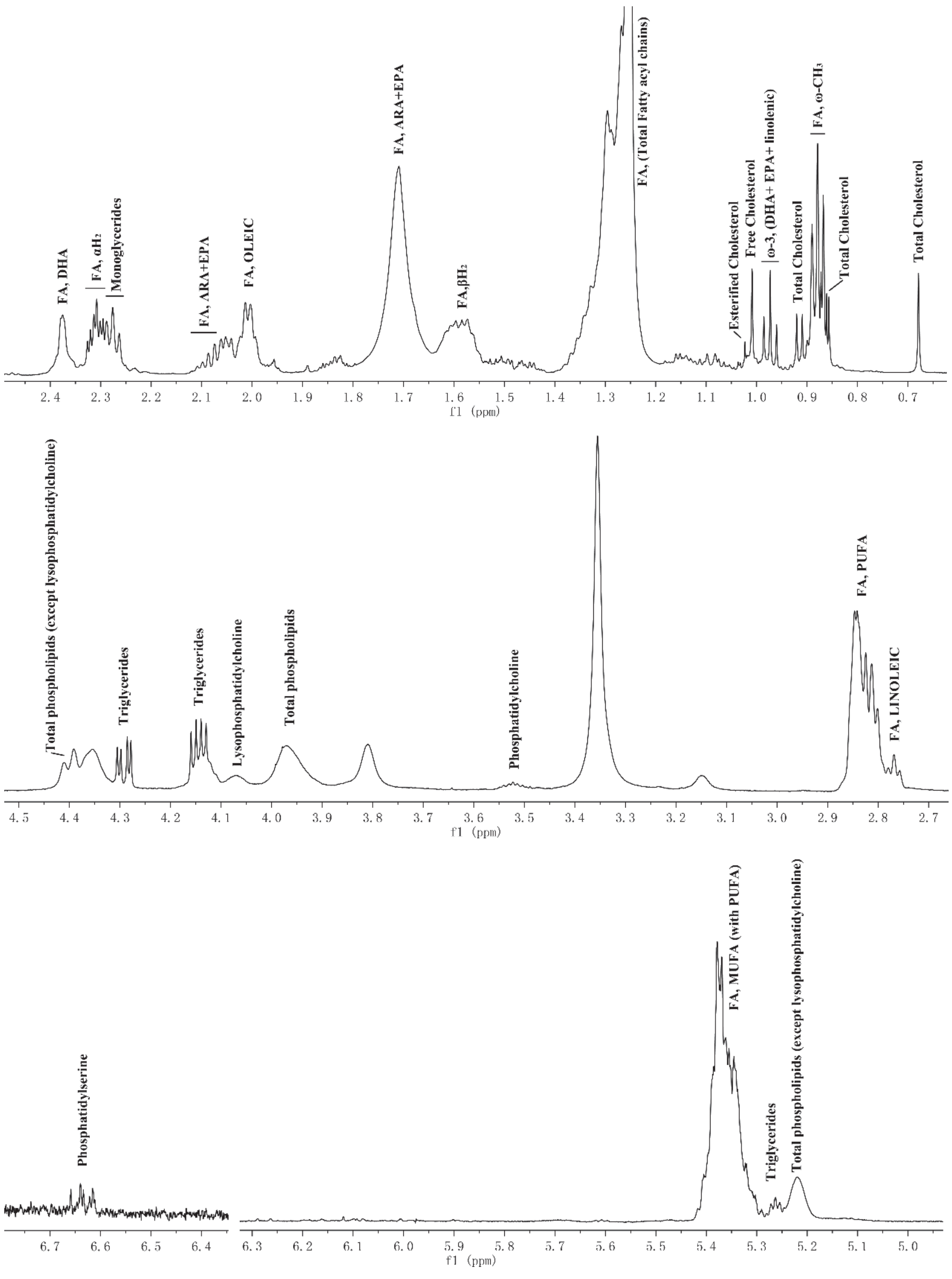

Figure S2. ^{1}H NMR spectra of liver chloroform extracts.

Table S1. List of metabolites assigned from NMR spectra.

Metabolites	^{1}H shift (ppm)	Moieties assignment
	**Water-soluble metabolites**	
Isoleucine	0.94(d) 1.01(d)	δCH_3, βCH_3
Leucine	0.96(t)	$\gamma CH_3+\gamma CH_3$
Valine	0.99(d) 1.04(d)	γCH_3
Methylsuccinate	1.09(d)	CH_3
Propylene glycol	1.14(d)	CH_3
Ethanol	1.19(t) 3.66(q)	βCH_3, CH_2
Lactate	1.33(d) 4.12(dd)	CH_3, CH
Alanine	1.48(d)	CH_3
Acetate	1.92(s)	CH_3
Glutamate	2.05(m) 2.12(m) 2.36(m)	Half βCH_2, Half βCH_2, γCH_2
Acetone	2.24(s)	CH_3
Glutamine	2.45(m)	γCH_2
Succinate	2.41(s)	$(CH_2)_2$
Dimethylamine	2.73(s)	CH3
Creatine	3.04(s) 3.93(s)	N-CH_3, CH_2
Acetylcarnitine	3.19(s)	$N(CH_3)_3$
Choline	3.20(s)	$N(CH_3)_3$
Phosphocholine	3.22(s) 3.59(m) 4.17(m)	$N(CH_3)_3$
Taurine	3.26(t) 3.42(t)	CH_2NH, CH_2SO_3
Trimethylamine-N-oxide (TMAO)	3.27(S)	$N(CH_3)_3$
Glucose	3.23-3.90(m)	CH
Glycine	3.56(s)	CH_2
Betaine	3.90(s)	NCH_2
Glycolate	3.95(S)	αCH
Inosine	4.28(dd) 4.44(t) 6.10(d) 8.23(s) 8.35 (s)	CH
UDP-glucose	7.96(d)	H6-ring
Fumarate	6.52(s)	CH=
Niacinamide	7.59(m) 8.25(m) 8.71(dd) 8.94(q)	CH
Adenine	8.19(s) 8.23(s)	N-CH=N
Formate	8.46(s)	HCOO-
	**Lipid-soluble metabolites**	
Total cholesterol	0.69(s) 0.86(2×d) 0.92(d)	C18-H_3, C26/27-H_3, C21-H_3
FA, ω-CH_3	0.88(t)	FA chain $CH_3(CH_2)_n$
ω-3,(DHA+EPA+Linolenic)	0.98(t)	ω-3 CH_3-CH_2-C=C
Free cholesterol	1.02(s)	C19-H_3
Esterified cholesterol	1.04(s)	C19-H_3
FA, (Total fatty acyl chains)	1.30(m)	FA chain -$(CH_2)_n$-
FA, βH_2	1.62(m)	βH_2 R-CH_2CH_2CO-OR
FA, ARA+EPA	1.70(m) 2.12(m)	βH_2 –CH=CH-CH_2-CH_2-CH_2CO-OR
		γH_2 –CH=CH-CH_2-CH_2-CH_2CO-OR
FA, OLEIC	2.02(m)	-CH=CH-CH_2-

Monoglycerides	2.29(t)	FA αH $-CH_2$-CO-O-C2
FA, αH_2	2.35(m)	αH_2 $-CH_2$-CO-OR
FA, DHA	2.41(m)	αH_2 & βH_2 -CHdCH-CH2-CH2-CO-OR
FA, LINOLEIC	2.78(t)	$-CH=CH-CH_2-(CH=CH-CH_2-)_n$, n=1
FA, PUFA	2.85(m)	$-CH=CH-CH_2-(CH=CH-CH_2-)_n$, n≥2
Phosphatidylcholine	3.54(m)	Alkyl-PC (Phosphoether) CH_2-N-$(CH_3)_3$
Total phospholipids	3.98(m)	Glycerol (C3-H_2)
Lysophosphatidylcholine	4.08(m)	C-1 CH_2-OC
Triglycerides	4.15(dd) 4.30(dd) 5.27(q)	Glycerol (C1-H^u) & (C3-H^u), Glycerol (C1-H^d) & (C3-H^d), Glycerol (C2-H)
Total phospholipids (except lysophosphatidylcholine)	4.40(m) 5.22(qd)	Glycerol (C1-H_2), Glycerol (C2-H)
FA, MUFA (with PUFA)	5.36(m)	-CH=CH-
Phosphatidylserine	6.64(m)	NH_3+ -CH-COO

Table S2. List of TOP 20 altered KEGG pathways by 0.050 mg/L TPhP exposure identified from transcriptomics analysis.

#	Pathway	DEGs with pathway annotation (333)	All genes with pathway annotation (21563)	Pvalue	Qvalue	Pathway ID
1	DNA replication	13 (3.9%)	54 (0.25%)	1.411347e-12	2.258155e-10	ko03030
2	Cell cycle	21 (6.31%)	216 (1%)	2.434798e-11	1.947838e-09	ko04110
3	Non-homologous end-joining	8 (2.4%)	25 (0.12%)	2.559716e-09	1.365182e-07	ko03450
4	Salivary secretion	19 (5.71%)	273 (1.27%)	5.582555e-08	2.233022e-06	ko04970
5	Oocyte meiosis	17 (5.11%)	230 (1.07%)	1.192191e-07	3.815011e-06	ko04114
6	Phagosome	28 (8.41%)	625 (2.9%)	5.280915e-07	1.408244e-05	ko04145
7	Progesterone-mediated oocyte maturation	13 (3.9%)	189 (0.88%)	8.140862e-06	1.756794e-04	ko04914
8	Fatty acid elongation	8 (2.4%)	67 (0.31%)	8.783972e-06	1.756794e-04	ko00062
9	Base excision repair	8 (2.4%)	71 (0.33%)	1.358167e-05	2.414519e-04	ko03410
10	Tuberculosis	23 (6.91%)	645 (2.99%)	0.0001892478	2.875599e-03	ko05152
11	p53 signaling pathway	11 (3.3%)	191 (0.89%)	0.0001976974	2.875599e-03	ko04115
12	Folate biosynthesis	4 (1.2%)	26 (0.12%)	0.0006383958	8.511944e-03	ko00790
13	Complement and coagulation cascades	11 (3.3%)	275 (1.28%)	0.003825453	4.708250e-02	ko04610
14	Proximal tubule bicarbonate reclamation	5 (1.5%)	72 (0.33%)	0.005124703	5.856803e-02	ko04964
15	Glutathione metabolism	5 (1.5%)	83 (0.38%)	0.00927622	9.894635e-02	ko00480
16	Renin-angiotensin system	3 (0.9%)	35 (0.16%)	0.01658405	1.658405e-01	ko04614
17	Fat digestion and absorption	5 (1.5%)	101 (0.47%)	0.02026400	1.907200e-01	ko04975
18	Glycosphingolipid biosynthesis - globo series	3 (0.9%)	41 (0.19%)	0.02524986	2.194395e-01	ko00603
19	Arginine and proline metabolism	6 (1.8%)	146 (0.68%)	0.02605844	2.194395e-01	ko00330
20	Amino sugar and nucleotide sugar metabolism	7 (2.1%)	196 (0.91%)	0.03299972	2.590526e-01	ko00520

Pathways with Q value < 0.05 are significantly enriched in DEGs.

Table S3. List of TOP 20 altered KEGG pathways by 0.300 mg/L TPhP exposure identified from transcriptomics analysis.

#	Pathway	DEGs with pathway annotation (264)	All genes with pathway annotation (21563)	Pvalue	Qvalue	Pathway ID
1	Phagosome	28 (10.61%)	625 (2.9%)	3.758622e-09	5.600347e-07	ko04145
2	Non-homologous end-joining	7 (2.65%)	25 (0.12%)	1.516482e-08	8.807107e-07	ko03450
3	DNA replication	9 (3.41%)	54 (0.25%)	1.773243e-08	8.807107e-07	ko03030
4	Oocyte meiosis	15 (5.68%)	230 (1.07%)	1.716864e-07	6.395318e-06	ko04114
5	Salivary secretion	16 (6.06%)	273 (1.27%)	2.849910e-07	8.492732e-06	ko04970
6	Cell cycle	14 (5.3%)	216 (1%)	4.797084e-07	1.191276e-05	ko04110
7	Progesterone-mediated oocyte maturation	12 (4.55%)	189 (0.88%)	3.945557e-06	8.398400e-05	ko04914
8	Base excision repair	7 (2.65%)	71 (0.33%)	2.596672e-05	4.836302e-04	ko03410
9	Tuberculosis	21 (7.95%)	645 (2.99%)	4.967419e-05	8.223838e-04	ko05152
10	Fatty acid elongation	6 (2.27%)	67 (0.31%)	0.000169935	2.532031e-03	ko00062
11	Complement and coagulation cascades	9 (3.41%)	275 (1.28%)	0.007018853	9.269175e-02	ko04610
12	PPAR signaling pathway	7 (2.65%)	184 (0.85%)	0.007698081	9.269175e-02	ko03320
13	Fat digestion and absorption	5 (1.89%)	101 (0.47%)	0.0080872	9.269175e-02	ko04975
14	Proximal tubule bicarbonate reclamation	4 (1.52%)	72 (0.33%)	0.01179087	1.254885e-01	ko04964
15	Glycosphingolipid biosynthesis - globo series	3 (1.14%)	41 (0.19%)	0.01373029	1.363875e-01	ko00603
16	Vitamin digestion and absorption	4 (1.52%)	98 (0.45%)	0.03244309	3.021263e-01	ko04977
17	Collecting duct acid secretion	3 (1.14%)	61 (0.28%)	0.0388304	3.403370e-01	ko04966
18	Mineral absorption	4 (1.52%)	111 (0.51%)	0.04766973	3.945994e-01	ko04978
19	Glycosphingolipid biosynthesis - ganglio series	2 (0.76%)	30 (0.14%)	0.05188978	4.069251e-01	ko00604
20	Glycosaminoglycan biosynthesis - keratan sulfate	2 (0.76%)	32 (0.15%)	0.05823893	4.338800e-01	ko00533

Pathways with Q value < 0.05 are significantly enriched in DEGs.

Table S4. Gene-specific primer sequences used for RT-qPCR.

Gene	Forward primer (5'–3')	Reverse primer (5'–3')	Tm (°C)
β-actin (NM_131031.1)	AGATGGGAACCGCTGCCTCTT	GTGGTCTCGTGGATACCGCAA	59
elovl7b (NM_199778.1)	GGCTAACGGCTACACCTAC	CCTCAGCACGAAGAACAC	59
faah2b (NM_001077462.1)	ACCCCTCTCACCCTCTTC	TCGCTCCTTACTCAACCC	59
fen1 (NM_198820.1)	CGGTTTATGTGTTTGATGG	TGCTCACCTGCTTCTTGT	59
cyp7a1a (NM_201173.2)	TTGCGCATGCTTTTGAACGA	TCAAAGGTTCGCCTCACCTC	59
tpi1b (NM_153668.4)	ATCGCCTGCATTGGTGAGAA	GAGCTTGTCATGCACCTCCT	59
cyp11a1 (NM_152953.2)	GAGGGCCATCACCCCAATAG	GTCTTCCAGGCCTTCCCTTC	59

Table S5. RT-qPCR validation of changes in selected genes identified by RNA-Seq.

Gene	RNA-Seq		RT-qPCR	
	0.050 mg/L	0.300 mg/L	0.050 mg/L	0.300 mg/L
elovl7b	-8.0	-4.1	-7.2±0.4	-5.1±0.3
faah2b	-8.9	-5.2	-6.9±0.2	-4.2±0.1
fen1	-7.4	-6.0	-7.0±0.3	-5.8±0.2
cyp7a1a	-2.6	-3.7	-2.2±0.4	-2.4±0.5
tpi1b	-6.1	-3.8	-5.5±0.6	-2.4±0.4
cyp11a1	-9.1	-5.4	-8.5±0.2	-6.0±0.3

Change of gene expression are expressed in log2(sample/control). Fold changes of these genes were similar by using these two inspect method, indicating that the RNA-Seq analysis was credible. Furthermore, since total mRNA samples used in RNA-Seq and RT-qPCR were collected from two parallel exposure, the similar result meaning that TPhP-induced transcriptomics alterations are inerratic.

Table S6. Change fold of specific DEGs mentioned in “discussion”.

DEGs	Full name	0.050 mg/L		0.300 mg/L		Related pathways (incomplete)	Category
		Fold change	Probability	Fold change	Probability		
CFTR8	ATP-binding cassette subfamily C member 8	-5.3	0.84	-1.0	0.12	Type II diabetes mellitus	Carbohydrate metabolism
INS	insulin	1.6	0.70	3.7	0.89	Type II diabetes mellitus	
tpiA	triosephosphate isomerase	-6.1	0.89	-3.8	0.81	Glycolysis / Gluconeogenesis	
sdhda	succinate dehydrogenase	-2.5	0.81	-5.5	0.85	Citrate cycle	
fuk	fucokinase	-8.1	0.87	-8.1	0.87	Fructose and mannose metabolism	
bdh	3-hydroxybutyrate dehydrogenase	-4.1	0.89	-2.8	0.80	Butanoate metabolism	
siat4a	sialyltransferase 4A	-8.7	0.90	-8.7	0.91	Glycan biosynthesis and metabolism	
gbgt1l4	globoside alpha-1,3-N-acetylgalactosaminyltransferase 1, like 4	-9.2	0.94	-3.9	0.85	Glycan biosynthesis and metabolism	
chiA	chitinase	-6.5	0.99	-7.7	0.99	Amino sugar and nucleotide sugar metabolism	
chs1	chitin synthase	-5.5	0.91	-3.2	0.80	Amino sugar and nucleotide sugar metabolism	
ppt	palmitoyl-protein thioesterase	-9.5	0.95	-3.3	0.83	Fatty acid metabolism	Lipid and fatty acid metabolism
elovl6	elongation of very long chain fatty acids protein 6	-5.2	0.89	-2.5	0.83	Fatty acid metabolism	
elovl7	elongation of very long chain fatty acids protein 7	-8.0	0.87	-4.9	0.83	Fatty acid metabolism	
cyp11a	cytochrome P450, family 11, subfamily A	-9.1	0.93	-5.4	0.90	Steroid hormone biosynthesis	
cyp7a1	cholesterol 7alpha-monooxygenase	-2.6	0.83	-3.7	0.88	Steroid hormone biosynthesis	
cyp17a1	cytochrome P450, family 17, subfamily A, polypeptide 1	-7.3	0.80	-2.1	0.61	Steroid hormone biosynthesis	
cyp1a2	cytochrome P450, family 1, subfamily A, polypeptide 2	1.0	0.66	3.3	0.88	Steroid hormone biosynthesis	
cyp2b	cytochrome P450, family 2, subfamily B	-0.2	0.27	2.0	0.80	Steroid hormone biosynthesis	
amiE	amidase	-8.9	0.92	-5.2	0.89	Arginine and proline metabolism	Amino acid metabolism
aoc1	diamine oxide	-8.1	0.88	-3.2	0.75	Arginine and proline metabolism	
dnmt1	DNA (cytosine-5)-methyltransferase 1	-3.5	0.84	-3.3	0.82	Cysteine and methionine metabolism	
ash1l	histone-lysine N-methyltransferase	-4.3	0.86	-4.6	0.86	Lysine degradation	

rrm1	ribonucleoside-diphosphate reductase subunit M1	-8.4	0.89	-5.1	0.86	Glutathione metabolism	Amino acid metabolism
anpep	amino peptidase N	-4.8	0.85	-2.1	0.69	Glutathione metabolism	
fmo5	flavin monooxygenase 5	-2.5	0.81	-2.8	0.83	Xenobiotics biodegradation and metabolism	TMAO metabolism
CHK2	serine/threonine-protein kinase	-4.6	0.83	-3.1	0.74	p53 signaling pathway	Signaling pathway
Scotin	scotin	-8.4	0.89	-3.3	0.77	p53 signaling pathway	
p53R2	ribonucleoside-diphosphate reductase subunit M2	-8.4	0.89	-5.1	0.86	p53 signaling pathway	
KAI	CD82 antigen	-8.4	0.90	-3.7	0.89	p53 signaling pathway	
ccnB	cyclin B	-10.7	0.97	-3.5	0.85	p53 signaling pathway	
ccnE	cyclin E	-5.2	0.87	-2.5	0.73	p53 signaling pathway	
Gadd45	growth arrest and DNA-damage-inducible protein	-0.8	0.60	-2.5	0.83	p53 signaling pathway	
FATCD36	CD36 antigen	-7.0	0.92	-6.1	0.91	PPAR signaling pathway	
FABP1	fatty acid-binding protein 1	-8.2	0.88	-8.2	0.87	PPAR signaling pathway	
LPL	lipoprotein lipase	-1.6	0.72	-3.6	0.85	PPAR signaling pathway	
PGAR	angiopoietin-like 4	3.2	0.88	3.7	0.90	PPAR signaling pathway	

Fold changes of DEGs are expressed in log2(sample/control).
